# Prevalence of Cytomegalovirus Antibodies in Croatian Childbearing-Aged and Pregnant Women: A Ten-Year Retrospective Study (2015–2024)

**DOI:** 10.3390/pathogens14090916

**Published:** 2025-09-11

**Authors:** Tatjana Vilibić-Čavlek, Klara Barbić, Tadej Ježek, Dan Navolan, Ana Sanković, Mario Sviben, Sara Glavaš, Daniel Mureșan, Laurentiu Pirtea, Maja Bogdanić

**Affiliations:** 1 Department of Virology, Croatian Institute of Public Health, 10000 Zagreb, Croatia; sara.glavas@hzjz.hr (S.G.); maja.bogdanic@hzjz.hr (M.B.); 2 School of Medicine, University of Zagreb, 10000 Zagreb, Croatia; tadej123jezek@gmail.com (T.J.); mario.sviben@hzjz.hr (M.S.); 3 Statistics Concentrator, Harvard University, Cambridge, MA 02139, USA; 4 Department of Obstetrics and Gynecology, “Victor Babes” University of Medicine and Pharmacy, 300041 Timisoara, Romania; navolan@umft.ro (D.N.); pirtea.laurentiu@umft.ro (L.P.); 5 Department of Microbiology, University of Applied Health Sciences, 10000 Zagreb, Croatia; asankovic@zvu.hr; 6 Department of Parasitology, Croatian Institute of Public Health, 10000 Zagreb, Croatia; 7 1st Department of Obstetrics and Gynecology, “Iuliu Haṭieganu” University of Medicine and Pharmacy, 400012 Cluj-Napoca, Romania; daniel.muresan@umfcluj.ro

**Keywords:** cytomegalovirus, seroprevalence, childbearing-aged women, pregnant women, Croatia

## Abstract

Due to possible congenital infections, cytomegalovirus (CMV) remains a significant public health concern in childbearing-aged and pregnant women. We analyzed the spatial, temporal, and age-related trends in CMV seroepidemiology in Croatia over 10 years. A total of 2838 childbearing-aged and pregnant women, aged 16–45 years, tested between 2015 and 2024 were included in the study. CMV IgM/IgG antibodies were detected using a commercial ELISA. IgM/IgG-positive samples were tested for IgG avidity. CMV IgG antibodies were detected in 2006 (70.6%) of participants. No significant differences were observed between 2015–2019 and 2020–2024 (72.0% vs. 69.8%), while yearly differences were of borderline significance, ranging from 62.4 to 77.3%. The overall seropositivity increased progressively with age from 49.6% in the 16–20 age group to 77.5% in the 36–40 age group. Significant regional differences in IgG seroprevalence were observed: 68.6% in the City of Zagreb/Northern Croatia, 78.5% in Pannonian Croatia, and 71.9% in Adriatic Croatia, while differences between settlement types were not significant. IgG seroprevalence was higher in women with an unfavorable obstetric history (85.3%) than in non-pregnant women and those with a normal pregnancy (70.6% and 66.5%, respectively). IgM antibodies were detected in 278 (9.8%) of participants. Acute infections were more common in younger participants, with rates decreasing from 13.6% in the youngest age group to 6.7% in the oldest. Logistic regression showed that age was a significant predictor of both IgG and IgM positivity. Region and obstetric history were significant predictors of IgG seropositivity, while settlement was a significant predictor of IgM seropositivity.

## 1. Introduction

Cytomegalovirus (CMV; human betaherpesvirus 5) is a ubiquitous DNA virus of the family *Orthoherpesviridae*. Like other herpesviruses, CMV establishes latency in hematopoietic progenitor cells in the bone marrow and myeloid progenitors after primary infection that can periodically reactivate [1]. CMV infections are common in both children and adults, with global seroprevalence rates ranging from 24 to 100% [2,3].

In healthy individuals, primary CMV infections are usually asymptomatic or present as a mild mononucleosis-like illness [4,5]. In immunocompromised patients, such as organ transplant recipients and HIV/AIDS patients, CMV is a leading cause of morbidity, causing more severe clinical symptoms due to the dissemination of the virus in various organs (pneumonia, retinitis, colitis, and esophagitis) [6]. Maternal CMV infection during pregnancy can lead to congenital infection [4].

CMV is transmitted through direct contact with infected bodily fluids, such as saliva, urine, genital secretions, and breast milk [7]. Pregnant women most often acquire the infection in contact with the saliva and urine of CMV-infected children, particularly their children [8]. The primary risk factor for maternal infection is exposure to young children under two years of age, who can shed the virus in their urine for up to 24 months [9].

CMV infections can manifest in three distinct forms: primary acute infections in seronegative individuals, reinfections with a different CMV strain despite pre-existing natural immunity, or, more commonly, reactivations of latent CMV infection. All these forms of infection can lead to vertical transmission [10].

With an estimated incidence of one in 200 infants in high-income environments, CMV is the most prevalent viral cause of congenital infections worldwide [11]. Primary CMV infections during pregnancy pose the highest risk of transplacental transmission [12]. CMV shedding in urine and cervicovaginal secretions increases as pregnancy advances, increasing the likelihood of transmission from 30–45% in the first trimester to 58–78% in the third trimester [13,14]. However, the likelihood of long-term sequelae for the fetus is significantly higher when infection occurs in the first trimester (24–26%) compared to infections acquired after 20 weeks of gestation (2.5–6%) [15,16].

The clinical manifestations of CMV infection differ based on the timing of infection (congenital, perinatal, or postnatal) [4]. In 85–90% of congenitally infected children, infection is asymptomatic; however, 10–15% of asymptomatic infants will later develop sensorineural hearing loss or learning disabilities [16]. Neonatal CMV infections are associated with a range of clinical manifestations, including intrauterine growth restriction, preterm birth, low birth weight, microcephaly, periventricular calcifications, chorioretinitis, petechiae, jaundice, hepatosplenomegaly, and sensorineural hearing loss [11].

The CMV seroprevalence in the general population differs regionally and is higher in lower socioeconomic groups. The seropositivity is up to 83% in developed countries, whereas in developing countries, the seroprevalence rate is nearly 100%. Among childbearing-aged women, CMV IgG seroprevalence ranged from 24.6% to 95.7%, aligning broadly with the estimated global seroprevalence in this group at 86% (2,3).

In Croatia, two studies conducted among women of childbearing age reported overall CMV seropositivity rates of 75.3% during 2005–2009 [17] and 70.5% during 2014–2023 [18]. However, temporal and spatial trends of CMV seroepidemiology in this population group were not analyzed. Given the observed declining trends in CMV seroepidemiology in several European countries, this study aimed to analyze the CMV seroprevalence trends among childbearing-aged and pregnant women in Croatia over 10 consecutive years.

## 2. Materials and Methods

### 2.1. Study Participants

**Figure 1:**
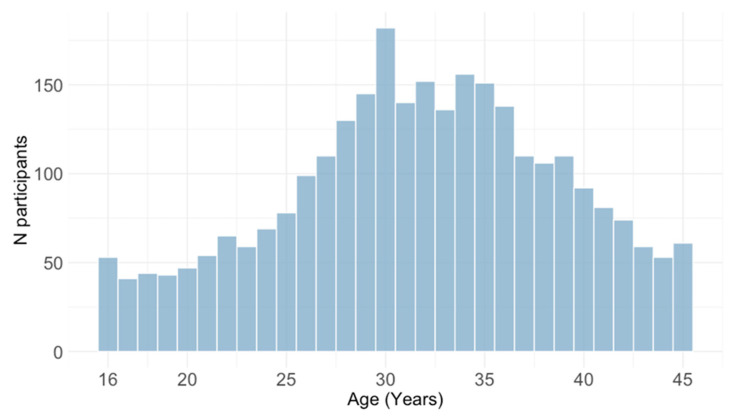
Age distribution of study participants.

The study included 2838 childbearing-aged and pregnant women aged 16–45 years (Figure 1) of Croatian nationality tested at the Croatian Institute of Public Health, the largest public health institution in the country. All women tested for CMV as part of the routine TORCH profile consecutively from January 2015 to December 2024 were analyzed. For this study, participants were classified by age (five-year age groups), residence area (urban or suburban/rural), geographic region, and obstetric history. Obstetric history included the categories of non-pregnant women, normal pregnancy, and unfavorable obstetric history: previous spontaneous abortions, children with congenital malformations, and infertility. The seroprevalence was analyzed in two 5-year periods (2015–2019 and 2020–2024) and yearly.

**Figure 2:**
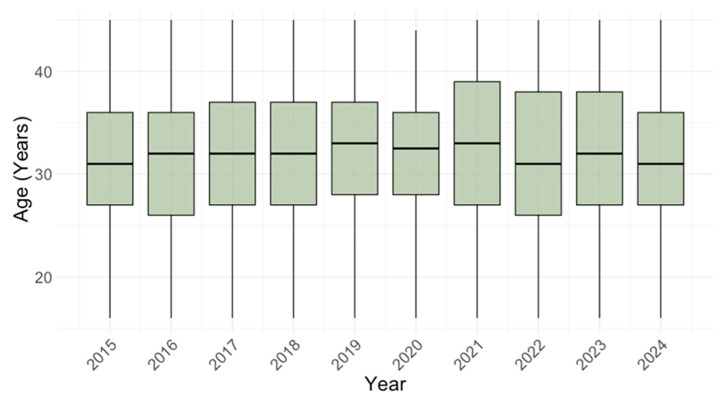
Median age of study participants by year.

There was no significant difference in the participants’ age between years of sampling (*p* = 0.203). The median age ranged from 31 years (IQR = 27–36) in 2015 to 33 years (IQR = 27–39) in 2021 (Figure 2).

To determine the seropositivity by geographic region, three regions were defined: City of Zagreb/Northern Croatia (*n* = 1761; 62.1%), Pannonian Croatia (*n* = 303; 10.7%), and Adriatic Croatia (*n* = 759; 26.7%) [19]. According to the settlement type, 2335 (82.3%) of participants were residents of urban regions, and 488 (17.2%) were residents of suburban/rural regions. For 15 (0.5%) of participants, data on the geographic region and settlement were missing. Data about the obstetric history were available for 2619 (92.3%) participants: 2264 (79.8%) were non-pregnant, 260 (9.2%) were pregnant, and 95 (3.3%) reported an unfavorable obstetric history (Table 1).

### 2.2. Methods

Initial serological screening for CMV-specific IgM and IgG antibodies was performed using a commercial automated enzyme-linked immunosorbent assay (ELISA; CMV IgG/CMV IgM Capture; Vircell Microbiologists, Granada, Spain). The antibody index was calculated and interpreted as follows: IgM/IgG < 9 negative, 9–11 equivocal, >11 positive. IgM/IgG-positive samples were additionally tested for IgG avidity (CMV Avidity; Euroimmun, Lübeck, Germany) to confirm or rule out recent CMV infection. IgG avidity was performed using urea as a denaturing agent, and the avidity index (AI) was calculated: AI < 40% low avidity (recent infection); 40–60% borderline avidity; >60% high avidity (previous infection).

### 2.3. Statistical Analysis

Descriptive statistics were used to summarize participant characteristics. CMV IgG and IgM seroprevalence was calculated overall and stratified by year, 5-year period, age group, region, settlement type, and obstetric history. Prevalence estimates were reported with 95% confidence intervals (CI) using binomial approximation. Differences between groups were assessed using Pearson’s chi-square test. Age distributions by serological status were visualized using boxplots. Multivariable logistic regression was used to identify predictors of IgG and IgM seropositivity, including age, year, region, settlement type, and obstetric history. Regression results were reported as log-odds estimates with 95% CI and visualized with coefficient plots. All analyses and visualizations were performed in R (version 4.4.2, R Foundation for Statistical Computing, Vienna, Austria), using the following packages: dplyr, ggplot2, tidyr, ggeffects, broom, patchwork, and kableExtra. A *p* < 0.05 was considered statistically significant.

## 3. Results

### 3.1. Temporal Trends of Cytomegalovirus IgG and IgM Seroprevalence

**Figure 3:**
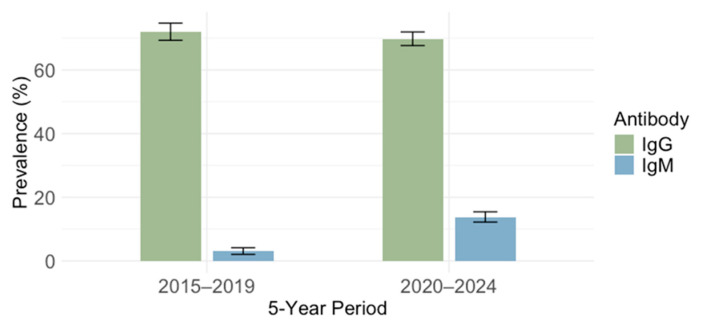
Prevalence of cytomegalovirus IgG and IgM antibodies in two periods: 2015–2019 and 2020–2024 (seroprevalence rates with 95% confidence intervals).

CMV IgG antibodies were found in 2006/2838 of participants (70.6; 95% CI: 68.9–72.3). Comparing the IgG seroprevalence in different 5-year periods, no significant difference (*p* = 0.210) in the seropositivity was observed in 2015–2019 (765/1062; 72.0%, 95% CI: 69.2–74.7) and 2020–2024 (1240/1776; 69.8%, 95% CI: 67.6–71.9) (Figure 3).

**Figure 4:**
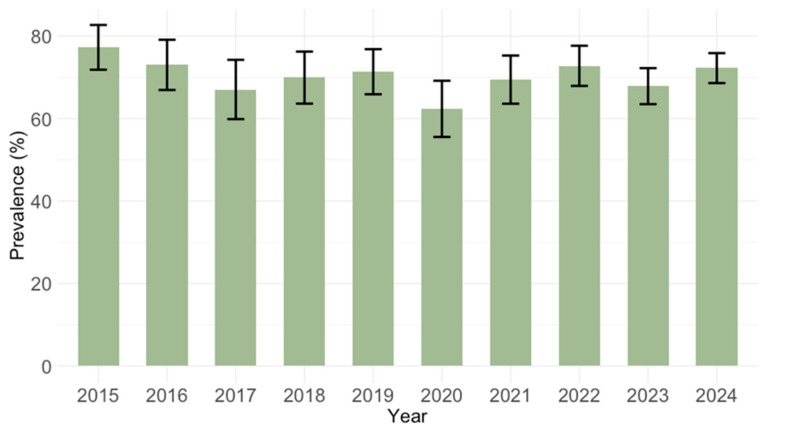
Prevalence of cytomegalovirus IgG antibodies by year (seroprevalence rates with 95% confidence intervals).

Analyzing the IgG prevalence by calendar year, the seropositivity varied from 62.4% (95% CI: 55.1–69.2) in 2020 to 77.3% (95% CI: 71.3–82.5) in 2015 (Figure 4). These differences did not reach statistical significance (*p* = 0.062).

**Figure 5:**
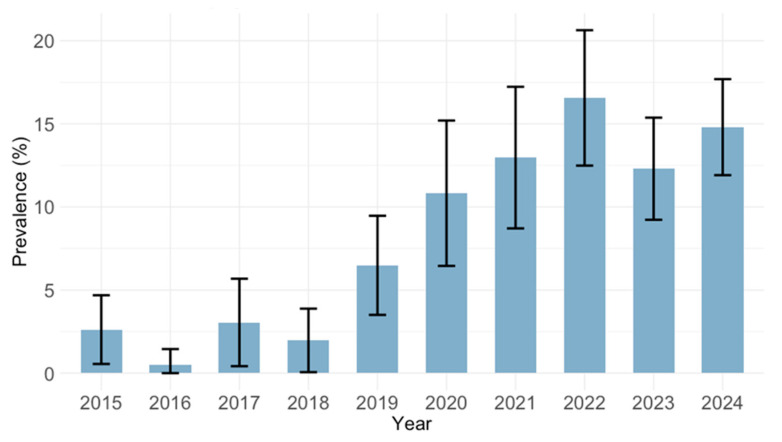
Prevalence of cytomegalovirus IgM antibodies by year (seroprevalence rates with 95% confidence intervals).

IgM antibodies were detected in 278 (9.8%; 95% CI: 8.7–10.9) participants, with significant differences between years of sampling (*p* < 0.001). The IgM positivity varied from 0.5% (95% CI: <0.01–2.7) in 2016 to 16.6% (95% CI: 12.7–21.1) in 2022 (Figure 5). Based on the results of IgG avidity in IgM/IgG positive samples, recent primary CMV infections were confirmed by low and borderline AI in 25 (0.8%; 95% CI: 0.6–1.3) patients, while in others (IgG antibodies of high avidity), detectable IgM antibodies indicated recurrent CMV infection.

### 3.2. Age-Related Trends of Cytomegalovirus IgG and IgM Seroprevalence

The overall CMV IgG and IgM seroprevalence (2015–2024) differed significantly among age groups (*p* < 0.001). The lowest IgG seropositivity was reported in the 16–20 age group (49.6%, 95% CI: 43.1–56%), followed by a progressive increase from 66.5% (95% CI: 61.2–71.4%) in 21–25-year-olds to 77.5% (95% CI: 73.9–80.8%) in 36–40-year-olds and remained stable thereafter. In contrast, acute infections (IgM positive) were more common in younger age groups, with a progressive decrease from 13.6% (95% CI: 9.7–11.7) in the 16–20 group to 6.7% (95% CI: 4.5–9.9) in the 41–45 group; however, these differences did not reach statistical significance (*p* = 0.090) (Table 2).

Significant differences in IgG seroprevalence rates by age were observed in both 5-year periods (*p* < 0.001). In 2015–2019, a continuous progressive increase in the seropositivity was observed from 53.3% (95% CI: 43.1–63.1) in the 16–20-year-olds to 81.1% (95% CI: 72.0–87.7) in the 41–45-year-olds. Between 2020 and 2024, seropositivity increased from 47.1% (95% CI: 38.9–55.4) in the 16–20 group to 72.2% (95% CI: 67.9–76.2) in the 31–35 group, with a stable rate thereafter. Comparing the same age groups in two time periods, no significant differences in the IgG seropositivity were found.

Comparing the IgM seroprevalence rates by age in two 5-year periods, significant differences were found in the first period (*p* = 0.010), while the second period did not reach statistical significance (*p* = 0.096). In 2015–2019, a decline in the IgM positivity was observed from 7.6% (95% CI: 3.7–14.9) in the 16–20-year-olds to 1.3% (95% CI: 0.4–3.6) in the 26–30-year-olds. In the following age groups, the seropositivity ranged from 2.1 (95% CI: 1.0–4.5) to 3.2% (95% CI: 1.1–8.9). In 2020–2024, IgM positivity ranged from 12.7 (95% CI: 9.5–16.7) to 17.6% (95% CI: 12.2–24.9) in participants aged 16–40 years, while it was lower in participants aged 41–45 years (8.2%; 95% CI: 5.3–12.4). Comparing the same age groups in two time periods, no significant differences in the IgM seropositivity were found.

**Figure 6:**
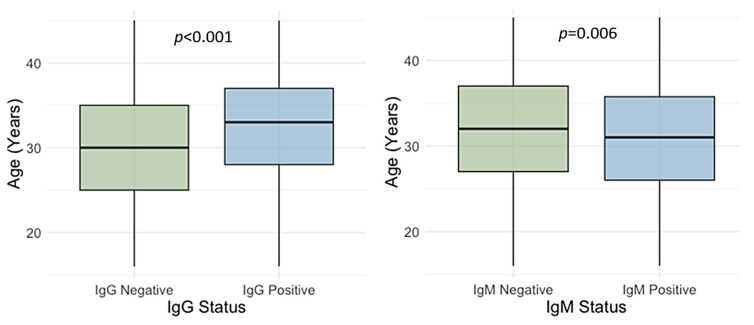
Age distribution of participants by cytomegalovirus IgG and IgM serological status. Horizontal lines in the boxes represent medians; boxes represent interquartile ranges; vertical lines represent ranges.

The participants’ age differed significantly between IgG-positive and IgG-negative individuals (*p* < 0.001) as well as IgM-positive and IgM-negative individuals (*p* = 0.006). The median age was higher in IgG-positive than in IgG-negative ones (median 33 years, IQR = 28–37 vs. 30 years, IQR = 25–35). In contrast, median age was lower in IgM-positive than in IgM-negative participants (median 31 years, IQR = 26–36 vs. 32 years, IQR = 27–37) (Figure 6).

### 3.3. Spatial Trends of Cytomegalovirus IgG and IgM Seroprevalence

**Figure 7:**
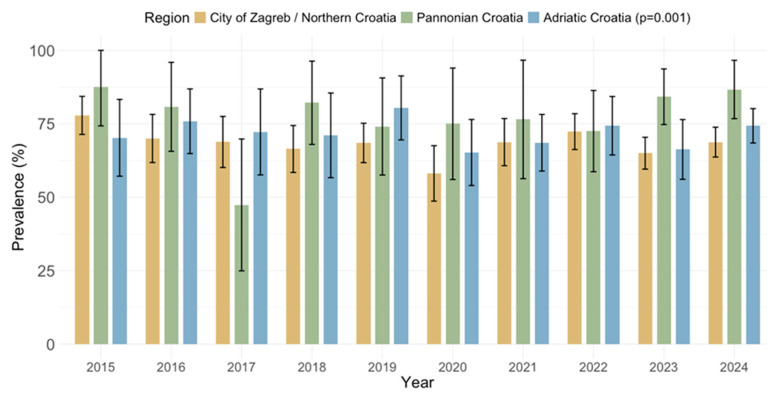
Prevalence of cytomegalovirus IgG antibodies by region (seroprevalence rates with 95% confidence intervals).

**Figure 8:**
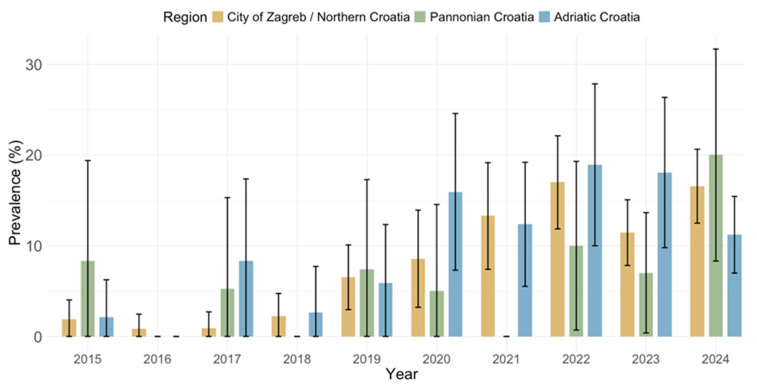
Prevalence of cytomegalovirus IgM antibodies by region (seroprevalence rates with 95% confidence intervals).

CMV IgG and IgM seroprevalence rates by geographic region are presented in Figure 7 and Figure 8. Significant differences in the IgG seropositivity were found between regions (*p* = 0.001): City of Zagreb/Northern Croatia 1208/1759 (68.6%; 95% CI: 66.4–70.8), Pannonian Croatia 238/303 (78.5%; 95% CI = 73.9–83.2), and Adriatic Croatia 546/759 (71.9%; 95% CI: 68.7–75.1) (Figure 7). However, the prevalence of IgM antibodies did not differ significantly between regions (*p* = 0.234): City of Zagreb/Northern Croatia 168; 9.6% (95% CI: 8.2–10.9), Pannonian Croatia 23; 7.6% (95% CI: 4.6–10.6), Adriatic Croatia 83; 10.9% (95% CI: 8.7–13.2 (Figure 8).

**Figure 9:**
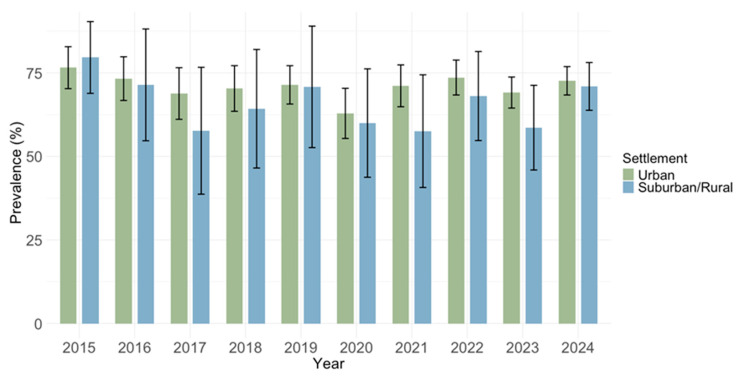
Prevalence of cytomegalovirus IgG antibodies by settlement type.

Analyzing the seroprevalence by settlement type, higher IgG seropositivity was found in urban areas (1663/2335; 71.2%; 95% CI: 69.4–73.1) compared to suburban/rural areas (329/488; 67.4%; 95% CI: 63.3–1.6); however, these differences did not reach statistical significance (*p* = 0.093) (Figure 9).

**Figure 10:**
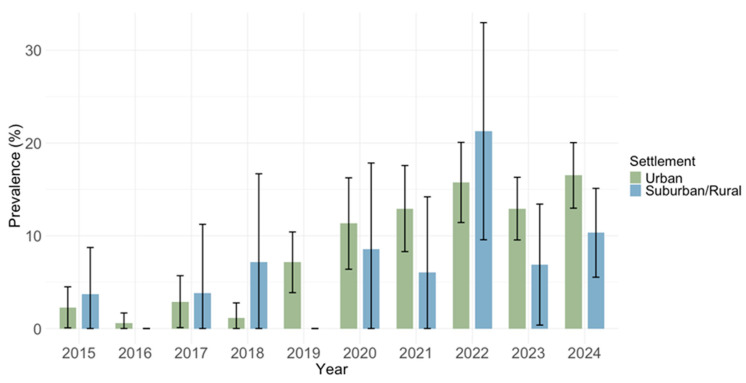
Prevalence of cytomegalovirus IgM antibodies by settlement type.

Prevalence of IgM antibodies did not differ by settlement (*p* = 0.215): urban areas 234/2335 (10.0%; 95% CI: 8.8–11.3) and suburban/rural areas 44/488 (9.0%; 95% CI: 6.6–11.9) (Figure 10).

### 3.4. Cytomegalovirus IgG and IgM Seroprevalence by Obstetric History

**Figure 11:**
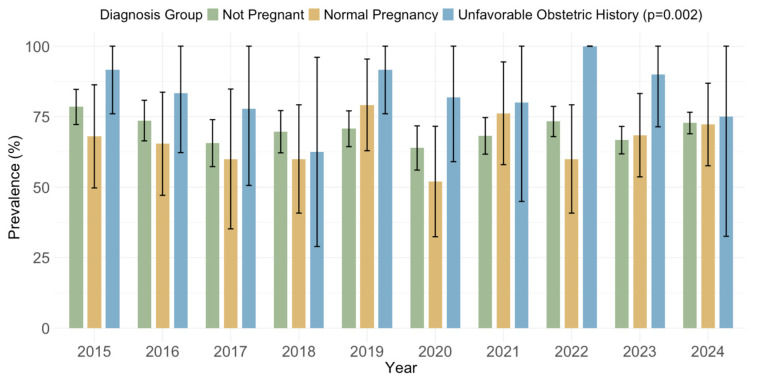
Prevalence of cytomegalovirus IgG antibodies by obstetric history (% positive with 95% confidence intervals).

Significantly higher overall IgG seroprevalence rates (*p* = 0.002) were observed in women with an unfavorable obstetric history (81/95; 85.3%; 95% CI: 78.1–92.4) than in non-pregnant women (1598/2261; 70.6%; 95% CI: 68.7–72.5) and women with normal pregnancies (173/260; 66.5%; 95% CI: 60.8–72.3) (Figure 11).

**Figure 12:**
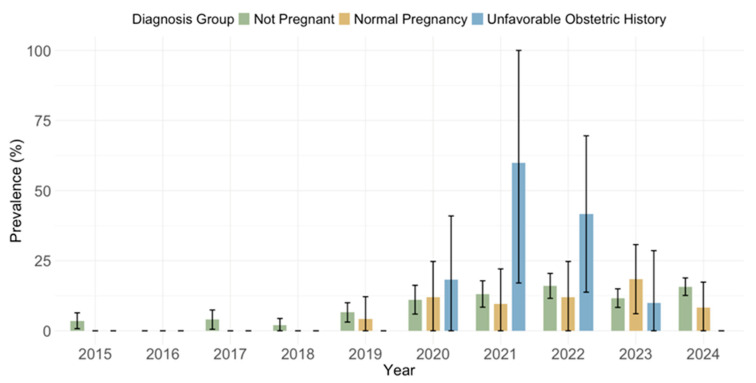
Prevalence of cytomegalovirus IgM antibodies by obstetric history (% positive with 95% confidence intervals).

The prevalence of acute infections (IgM positive) did not differ by diagnosis (*p* = 0.263): non-pregnant 235; 10.4% (95% CI: 9.1–11.7), normal pregnancy 19; 7.3% (95% CI: 4.1–10.5), unfavorable obstetric history 11; 11.6% (95% CI: 5.1–18.0) (Figure 12).

### 3.5. Risk Analysis for Cytomegalovirus IgG and IgM Seropositivity

A linear regression analysis revealed that IgG-seropositive individuals were, on average, 2.3 years older than those who were seronegative (β = 2.2, 95% CI: 1.7–2.8). This difference was statistically significant (*p* < 0.001). In contrast, IgM-seropositive individuals were, on average, 1.3 years younger than those who were seronegative (β = −1.29, 95% CI: −2.16–−0.41). This difference was also statistically significant (*p* = 0.004).

**Figure 13:**
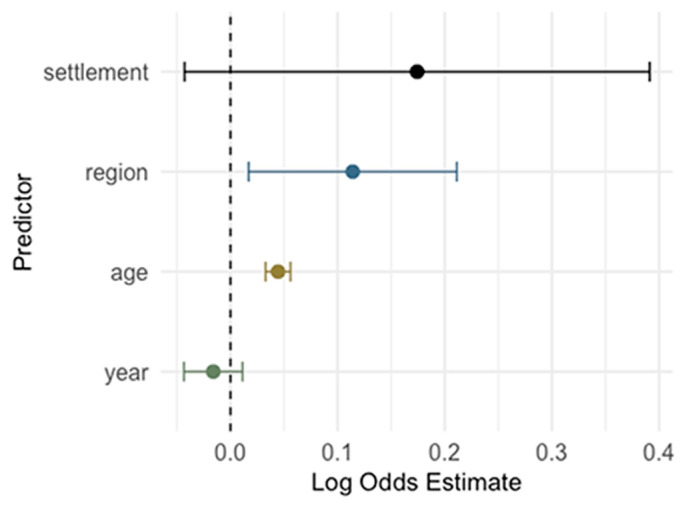
Logistic regression analysis for the risk of cytomegalovirus IgG seropositivity (log odds with 95% confidence intervals; CI). A coefficient > 0 means the predictor is associated with increased odds of being IgG-positive, and a coefficient < 0 means decreased odds of being positive. Predictors whose CI exclude 0 are statistically significant.

A logistic regression model was used to evaluate the association between demographic variables (year of sampling, age, region, and settlement type) and IgG/IgM seropositivity (Figure 13). Age was a significant predictor: each additional year of age was associated with increased odds of IgG seropositivity (OR = 1.05, 95% CI: 1.03–1.06, *p* < 0.001), suggesting older individuals were more likely to be IgG seropositive. Compared to participants from Zagreb/Northern Croatia, those from Pannonian Croatia had significantly higher odds of IgG seropositivity (OR = 1.69, 95% CI: 1.26–2.26, *p*< 0.001). The difference between Adriatic Croatia and Zagreb/Northern Croatia did not reach statistical significance (OR = 1.18, 95% CI: 0.98, 1.42, *p* = 0.085). Year and settlement type were not statistically significant predictors in this model (*p* = 0.250 and *p* = 0.120, respectively), indicating no temporal trends or differences between urban and rural settings once age and region were accounted for.

**Figure 14:**
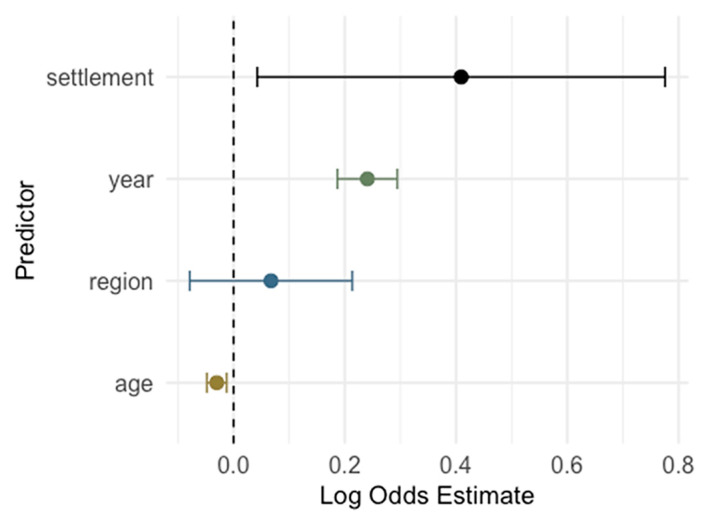
Logistic regression analysis for the risk of cytomegalovirus IgM seropositivity (log odds with 95% confidence intervals; CI). A coefficient > 0 means the predictor is associated with increased odds of being IgM-positive, and a coefficient < 0 means decreased odds of being positive. Predictors whose CI exclude 0 are statistically significant.

Year was a strong and significant predictor for IgM positivity: each additional calendar year was associated with higher odds of IgM seropositivity (OR = 1.27, 95% CI: 1.18–1.37; *p* < 0.001), indicating a clear upward trend in IgM prevalence over time. Age showed a significant inverse association: younger individuals were more likely to be IgM positive (OR = 0.97 per year increase in age, 95% CI: 0.95–0.99, *p* < 0.001), suggesting recent infections occur more often in younger individuals. Settlement type (urban vs. suburban/rural) was also a significant factor (*p* = 0.029), with urban participants having higher odds of IgM positivity (OR = 1.51). Region was not a significant predictor in the adjusted model (*p* = 0.370), indicating no strong regional differences in IgM prevalence after adjusting for other factors (Figure 14).

A logistic regression model was used to evaluate the association between diagnosis group (non-pregnant, normal pregnancy, unfavorable obstetric history) and IgG/IgM seropositivity, adjusting for year of sampling (Table 3). Compared to participants who were not pregnant, those with an unfavorable obstetric history had significantly higher odds of IgG seropositivity (OR = 2.46, 95% CI = 1.38–4.39; *p* = 0.002). No statistically significant difference was found between normal pregnancies and non-pregnant participants (OR = 0.84, 95% CI = 0.64–1.11; *p* = 0.216). Several calendar years were associated with significantly lower odds of IgG seropositivity compared to 2015, particularly 2017 (*p* = 0.011), 2018 (*p* = 0.033), 2020 (*p* = 0.001), and 2023 (*p* = 0.009), indicating a potential temporal decline in IgG prevalence. Compared to participants who were not pregnant, no statistically significant differences in IgM seropositivity were observed among those with normal pregnancies (OR = 0.80, *p* = 0.365) or unfavorable obstetric history (OR = 1.62, *p* = 0.162). In contrast, the sampling year showed strong temporal associations. Relative to 2015, significantly higher odds of IgM seropositivity were observed in 2020 (*p* = 0.001), 2021 (*p* < 0.001), 2022 (*p* < 0.001), 2023 (*p* < 0.001), and 2024 (*p* < 0.001). These results indicate a temporal increase in acute CMV infections (IgM seropositivity) over time, while no significant differences were attributable to pregnancy status.

## 4. Discussion

The CMV seroprevalence dynamic is changing. Due to the risk of congenital infection, CMV remains a significant public health concern in childbearing-aged and pregnant women. Therefore, it is important to define the seronegative women who are at risk of primary infection during pregnancy.

The results of this study showed an overall IgG seroprevalence of 70.6%, which is lower than the 75.3% reported in a previous Croatian study conducted between 2005 and 2009 [17]. Similar declining trends in the seroprevalence were observed in several European countries. A cross-sectional study conducted in Finland at three time points (1992, 2002, and 2012) showed that CMV seroprevalence decreased significantly over 20 years, from 84.5% in 1992 to 71.5% in 2012 [20]. In a large-scale study from western Romania, the CMV seropositivity of pregnant women decreased significantly from 2008–2010 (94.68%) to 2015–2018 (91.80%) [21]. The declining CMV seroprevalence among women of childbearing age, especially in high-income countries, can be attributed to several factors, including better sanitation, smaller households, and less crowding, which reduce early childhood exposure [4,14,22].

In contrast, there was no change in the overall seroprevalence in childbearing-aged women in Germany, with CMV seropositivity of 64.18% in the decade 1988–1997 and 65.95% in 2009–2018 [23]. A stable trend of CMV IgG seroprevalence over five years (2019–2023), ranging from 62% to 66%, was observed in Italy [24]. In addition, stable but higher seroprevalence rates were found in southwest Romania (93.68% seropositivity in 2013–2016 and 94.96% in 2019–2022, respectively) [25]. In France, the seroprevalence among pregnant women increased from 52.7% during 2017–2019 to 58.4% in the period 2020–2022 [26].

Comparing the CMV seropositivity in Croatia with other European countries, similar seroprevalence rates to Croatia were found in Italy (70.8–72.1%) [27,28]. Higher seropositivity was reported in Bulgaria (84.7%) [29], Bosnia and Herzegovina (89.6–96.4%) [30], and Kosovo and Metohija (96.2%) [31], and lower in Greece (66.49%) [32], Spain (62.2%) [33], Norway (54%) [34], and France (45.6%) [35]. In Poland, seroprevalence varied widely, ranging from 63.7% [14] to 81.9% [36]. Similarly, the seroprevalence in Turkey ranged from 35.52% [37] to 98.8% [38]. In the United Kingdom (UK), differences in seropositivity were observed among pregnant women by ethnic group. CMV seroprevalence was observed at 49% in White British women, 89% in South Asian women born in the UK, and 98% in South Asian women born in South Asia [39]. A very low seroprevalence was found in Irish pregnant women. Only 30.4% were CMV antibody positive compared to 89.7% of non-Irish women [40]. Similarly, low seroprevalence was found in Belgium (30%) [22].

Some countries showed spatio-temporal regional differences in CMV IgG seroprevalence within the country. In Poland, the seroprevalence was 76.7% in central Poland in 1999–2009 [41] and 63.7% in the Pomeranian and Kujavian–Pomeranian regions in 2003–2023 [14]. In the Croatian study, geographic region was associated with CMV seropositivity, with the highest rate observed in Pannonian Croatia (78.5%) compared to the City of Zagreb/Northern Croatia and Adriatic Croatia (68.6 and 71.9%, respectively). A higher seroprevalence in Pannonian Croatia can be attributed to a combination of socioeconomic, demographic, and environmental factors. The population of the Pannonian regions is mostly rural and agricultural. Higher birth rates and earlier parenthood in rural areas can lead to more early-life CMV exposure, especially from young children shedding the virus. Close and frequent contact with young children in multigenerational households further increases the seroprevalence. Differences in housing quality, sanitation, and urbanization between Pannonian Croatia and more urbanized Adriatic Croatia can also contribute to the CMV transmission dynamic.

The presented study showed an age-related overall and yearly CMV IgG seroprevalence. A progressive increase in seropositivity with age was observed from 49.6% in the 16–20 age group to 77.5% in the 36–40 age group. In contrast, the prevalence of acute infections (IgM positive) declined from 13.6% in the 16–20 group to 6.7% in the 41–45 group, reflecting the higher proportion of non-immune women in younger age groups. Age was also a significant predictor for IgG seropositivity in logistic regression, with each additional year of age associated with increased odds of IgG seropositivity, suggesting older individuals were more likely to be CMV IgG seropositive. In contrast, age showed a significant inverse association with IgM seropositivity. Younger individuals were more likely to be IgM positive, suggesting recent infections occur more frequently at a younger age.

Similar age-related increasing trends were observed in some other countries, including Bosnia and Herzegovina [30], Poland [36], and France [35]. The increase in CMV seroprevalence with age reflects the cumulative exposure to the virus over time in various settings (home, school, and workplace). Once infected, people remain seropositive for life, so the proportion of seropositive individuals naturally increases with age. In Germany, young pregnant women aged 15 to 25 years showed a higher seropositivity rate compared to older pregnant women [13]. In Romania, seroprevalence rates were consistently high across all groups (92.5–100%), with no significant association observed between maternal age and seroprevalence [21]. No significant differences among age groups were also observed in Finland [20] and Norway [34].

Analyzing the relationship between the settlement type and CMV seroprevalence, our study found a borderline significant association, with urban participants showing only slightly higher odds of IgG positivity compared to rural ones. Several factors can explain the higher seroprevalence in urban areas. Urban environments have more crowded schools, daycares, and housing. Due to higher population density, interpersonal contacts are more frequent, increasing the spread of the virus. Additionally, working parents in urban areas are more likely to use group childcare, where CMV spreads easily among toddlers. CMV seroprevalence is higher in individuals with lower socioeconomic status [3]. Urban regions often have higher levels of poverty and housing insecurity, which are linked to earlier and more frequent CMV exposure. Furthermore, many urban centers have larger immigrant populations from regions with higher CMV seroprevalence (e.g., Africa, Asia, and Latin America). The inconsistent results regarding settlement were observed in Romania. In one study, the seroprevalence was higher in rural areas compared to urban areas [25]. In another study, the difference in the CMV seropositivity between urban and rural settlements was not significant in 2008–2010 (94.50 vs. 95.33%), while it was significant in 2015–2018, with higher seropositivity in rural areas (94.92 vs. 89.08%) [21].

Analyzing the seroprevalence by obstetric history, this study found significantly higher IgG seropositivity in women with an unfavorable obstetric history (85.3%), compared to non-pregnant women (70.6%) and women with normal pregnancies (66.5%). These results were confirmed by logistic regression, showing higher odds in women with an unfavorable obstetric history. However, no differences were observed for IgM seropositivity. In a previous Croatian study (2013–2015), no difference was observed between groups with normal pregnancy (78.2% IgG seropositive) and an unfavorable obstetric history (78.1% IgG seropositive) [42]. Higher IgM and IgG seroprevalence in women with an unfavorable obstetric history was observed in some studies [43,44]. A strong but borderline significant association between the presence of latent CMV infection (IgG seropositive) and the history of spontaneous abortion was found in Romania [45]. In Bosnia and Herzegovina, CMV IgG seroprevalence was high among women regardless of abortion history, with rates of 94.5% in those with previous abortions and 91.9% in those without [30]. Women with higher CMV seroprevalence may have had subclinical or symptomatic infections that contributed to unfavorable obstetric outcomes.

Although CMV IgM antibodies were detected in 9.8% of participants, only 0.8% showed a low IgG avidity suggestive of primary infection. In the others, positive IgM antibodies may indicate recurrent infections (reactivations or reinfections). In addition, false-positive IgM antibodies can occur due to cross-reactivity with other viral infections (most commonly herpes simplex virus, varicella-zoster virus, and Epstein-Barr virus) or as a result of interference from autoimmune antibodies like rheumatoid factor [46].

This study has several limitations that should be addressed. Its retrospective design, relying on previously collected laboratory data, restricts access to detailed individual-level information, such as socioeconomic status or occupation that could influence variations in seroprevalence. Furthermore, as the study was conducted at a national reference center, individuals utilizing private healthcare services or those from marginalized populations may have been underrepresented, potentially introducing selection bias.

CMV seroprevalence varies between lower and higher socioeconomic groups [3], a factor that was not analyzed in this study. While this study focused on unfavorable obstetric history, it is also important to consider whether other underlying conditions, such as immunosuppressive disorders, may influence the risk of CMV infection.

Serology screening was conducted using ELISA; however, no confirmatory immunoblot testing was performed on IgM-positive samples, which may have contributed to the high rate of IgM positivity. Lastly, there is a risk of misclassification due to equivocal or inconclusive serology, particularly in the absence of follow-up testing.

## 5. Conclusions

This study provides important insights into the CMV seroepidemiology in the risk population group, such as childbearing-aged and pregnant women. The study highlights temporal and age-related trends in CMV seroprevalence that may inform public health for planning and targeted interventions. Although routine CMV testing is usually not recommended for all childbearing-aged women, targeted testing and education are important tools in reducing risks for congenital CMV infections. Information about the CMV serological status can help seronegative women to take preventive measures and allow for early diagnosis and management of infected pregnant women.

## Figures and Tables

**Table 1:** Distribution of participants according to demographic characteristics, area of residence, and obstetric history.

Characteristic		N (%) Tested
Age group	16–20 years	228 (8.0)
	21–25 years	325 (11.5)
	26–30 years	666 23.5)
	31–35 years	735 (25.9)
	36–40 years	556 (19.6)
	41–45 years	328 (11.5)
Geographic region	City of Zagreb/Northern Croatia	1761 (62.1)
	Pannonian Croatia	303 (10.7)
	Adriatic Croatia	759 (26.7)
	Missing	15 (0.5)
Settlement	Urban	2335 (82.3)
	Suburban/rural	488 (17.2)
	Missing	15 (0.5)
Obstetric history	Non-pregnant	2261 (79.8)
	Pregnant	260 (9.2)
	Unfavorable obstetric history	95 (3.3)
	Missing	219 (7.7)

**Table 2:** Cytomegalovirus IgG and IgM seropositivity by age.

Age Group	Tested	CMV IgG		*p*	CMV IgM		*p*
	N	N (%)	95% CI		N (%)	95% CI	
16–20 years	228	113 (49.6)	43.1–56.0	<0.001	31 (13.6)	9.7–11.7	0.090
21–25 years	325	216 (66.5)	61.2–71.4		38 (11.7)	8.6–16.5	
26–30 years	666	457 (68.6)	65.0–72.0		67 (10.1)	8.0–12.6	
31–35 years	735	537 (73.1)	69.7–76.1		72 (9.8)	7.9–12.2	
36–40 years	556	432 (77.5)	73.9–80.8		48 (8.6)	6.6–11.3	
41–45 years	328	251 (76.5)	71.6–80.8		22 (6.7)	4.5–9.9	
Total	2838	2006 (70.6)	68.9–72.3		278 (9.8)	8.7–10.9	

CI = confidence interval.

**Table 3:** Risk analysis for cytomegalovirus IgM and IgG seropositivity according to obstetric history, adjusting for year.

Year	CMV IgG Odds Estimate	*p*	CMV IgM Odds Estimate	*p*
2015	Ref.		Ref.	
2016	−0.266	0.263	−14.066	0.961
2017	−0.618	0.011	0.130	0.831
2018	−0.499	0.033	−0.557	0.435
2019	−0.285	0.203	0.743	0.135
2020	−0.719	0.001	1.476	0.001
2021	−0.434	0.051	1.685	<0.001
2022	−0.250	0.242	1.896	<0.001
2023	−0.519	0.009	1.543	<0.001
2024	−0.256	0.186	1.800	<0.001

## Data Availability

The original contributions presented in the study are included in the article; further inquiries can be directed to the corresponding author.

## Abbreviations

The following abbreviations are used in this manuscript:

CMV	Cytomegalovirus
TORCH	Toxoplasma, other, rubella, cytomegalovirus, herpes simplex virus
HIV	Human immunodeficiency virus
AIDS	Acquired immunodeficiency syndrome
AI	Avidity index
CI	Confidence interval
IQR	Interquartile range
